# Impact of Preoperative Ultrasound-Guided Rectus Sheath Block on Postoperative Recovery After Robot-Assisted Gynecologic Surgery: A Retrospective Cohort Study

**DOI:** 10.3390/jcm15135034

**Published:** 2026-06-28

**Authors:** Hwa-Young Jang, Yun Choi, Chang-Woo Kim, Jeongmin Gu, Yoonhee Choi, Sang-Wook Lee, Sung-Hoon Kim, Ji-Yeon Sim

**Affiliations:** 1 Department of Anesthesiology and Pain Medicine, Asan Medical Center, University of Ulsan College of Medicine, Seoul 05505, Republic of Korea; janghy315@amc.seoul.kr (H.-Y.J.); d251226@amc.seoul.kr (J.G.); is00298@amc.seoul.kr (Y.C.); sangwooklee20@gmail.com (S.-W.L.); 2 College of Medicine, Seoul National University, Seoul 03080, Republic of Korea; daysinthesunava@gmail.com; 3 Department of Biomedical Engineering, University of Ulsan College of Medicine, Seoul 05505, Republic of Korea; kcw.amc@gmail.com

**Keywords:** rectus sheath block, robot-assisted gynecologic surgery, multimodal analgesia, enhanced recovery after surgery, length of hospital stay, postoperative pain

## Abstract

**Background/Objectives**: Postoperative pain after robot-assisted gynecologic surgery delays recovery and prolongs hospitalization, yet evidence on the role of ultrasound-guided rectus sheath block (RSB) in this setting is limited. We investigated whether preoperative ultrasound-guided RSB was associated with a shorter length of hospital stay (LOS) after robot-assisted gynecologic surgery. **Methods**: This single-center retrospective cohort study included 266 consecutive female patients who underwent robot-assisted gynecologic surgery between November 2023 and April 2024. RSB was introduced in January 2024; 113 patients from the RSB-eligible era who received the block and 153 patients before RSB introduction served as the RSB and comparator groups, respectively. The primary outcome was LOS. Secondary outcomes included rescue intravenous fentanyl and rescue antiemetic use in the post-anesthesia care unit (PACU) and rescue analgesic administration on the general ward through postoperative day (POD) 2. Multivariable quasi-Poisson regression was used to adjust for potential confounders. **Results**: LOS was significantly shorter in the RSB group (median 3 [IQR 3–3] vs. 4 [3–5] days; *p* < 0.001; adjusted IRR 0.78, 95% CI 0.72–0.85). The RSB group also required less PACU rescue fentanyl (0.86 [0.68–1.38] vs. 1.17 [0.85–1.69] μg/kg; *p* < 0.001), fewer rescue antiemetics (3.5% vs. 11.8%; *p* = 0.029), and fewer ward rescue analgesics on POD 0 (52.2% vs. 68.6%; *p* = 0.009) and POD 1 (13.3% vs. 34.2%; *p* < 0.001). **Conclusions**: Preoperative ultrasound-guided RSB was associated with shorter LOS, reduced PACU opioid and antiemetic requirements, and fewer early ward rescue analgesics. Randomized trials are warranted to confirm these benefits.

## 1. Introduction

Robot-assisted surgery has been increasingly adopted in gynecology over the past decade [1,2]. Compared with open surgery, the robotic approach has been associated with reduced intraoperative blood loss, fewer perioperative complications, and shorter hospital stay [1,3]. Nevertheless, the periumbilical camera port and multiple lateral working ports remain sources of clinically significant somatic pain, and most patients still require opioid analgesia in the immediate postoperative period [3,4]. Inadequately controlled postoperative pain delays ambulation, increases the risk of opioid-related adverse effects, and prolongs hospitalization—all of which run counter to the goals of enhanced recovery after surgery (ERAS) protocols for gynecologic surgery [5].

Multimodal analgesia incorporating regional techniques is a cornerstone of contemporary ERAS pathways [5,6]. Among truncal blocks, the ultrasound (US)-guided rectus sheath block (RSB) provides somatic analgesia of the periumbilical region and midline abdominal wall. Because the umbilicus is the principal entry site for the camera port in most robotic gynecologic procedures, the sensory distribution of RSB is anatomically well matched to the typical pattern of postoperative wound pain in this population. Randomized trials and meta-analyses have demonstrated that RSB reduces early postoperative pain scores and opioid consumption after laparoscopic abdominal and gynecologic surgery [7,8,9].

In contrast, evidence supporting RSB specifically in robot-assisted gynecologic surgery is sparse. A prior single-arm retrospective case series examined 20 patients in which all participants received postoperative RSB without a comparator group, and length of hospital stay (LOS) was not assessed [10]. The comparative effect of US-guided RSB versus standard care on postoperative recovery after robot-assisted gynecologic surgery remains unestablished.

We therefore conducted a retrospective cohort study to investigate whether the addition of preoperative US-guided RSB to a standardized multimodal analgesic regimen would shorten the LOS after robot-assisted gynecologic surgery. LOS was selected as the primary outcome because it represents an integrative measure of recovery and a clinically meaningful endpoint within ERAS frameworks. Secondary outcomes included opioid consumption in the post-anesthesia care unit (PACU), the occurrence of postoperative nausea and vomiting (PONV) in the PACU, and analgesic requirements on the general ward through postoperative day (POD) 2.

## 2. Materials and Methods

### 2.1. Study Population

This retrospective cohort study was conducted using electronic medical records at Asan Medical Center, Seoul, Republic of Korea. Consecutive female patients who underwent robot-assisted gynecologic surgery using the da Vinci Si, Xi, or SP^®^ system (Intuitive Surgical, Sunnyvale, CA, USA) between November 2023 and April 2024 were screened for eligibility. Inclusion criteria were: (1) female sex; (2) age ≥ 18 years; (3) elective robot-assisted gynecologic surgery; and (4) completion of a standard PACU course prior to ward transfer. Exclusion criteria were: (1) direct transfer to the intensive care unit without completing a standard PACU course; (2) incomplete perioperative records; (3) RSB performed after surgical completion rather than before incision; and (4) active refusal of RSB after preoperative consultation.

US-guided RSB was introduced as an optional component of the institutional multimodal analgesia protocol in January 2024. After this date, patients scheduled for robot-assisted gynecologic surgery were routinely informed about RSB during preoperative consultation. Study cohort assignment was defined by surgical era: the RSB group comprised patients from the RSB-eligible era who received the block, and the non-RSB group comprised those who underwent surgery during the pre-RSB era. The study protocol was approved by the Institutional Review Board of Asan Medical Center (protocol no. 2024-1080), which waived the requirement for written informed consent given the retrospective nature of the study. Data collection was conducted from October 2024 to January 2025, and data analysis was completed in May 2025. The study was conducted in accordance with the Declaration of Helsinki and its subsequent amendments.

### 2.2. General Anesthesia and Pain Control

All robotic procedures during the study period were performed by three fully trained attending gynecologic surgeons whose practice patterns did not change, and the institutional anesthesia protocol, postoperative analgesic regimen, and discharge criteria remained unchanged throughout the study period; the only relevant change was the introduction of optional RSB in January 2024.

General anesthesia was induced with propofol (2 mg/kg), rocuronium (0.6 mg/kg), and a target-controlled infusion of remifentanil targeting an effect-site concentration of 2–5 ng/mL. Anesthesia was maintained with desflurane 5–6% in 50% oxygen, with the vaporizer titrated within ±1.0 vol% to keep the bispectral index between 40 and 60. The remifentanil was continued throughout the operation, and the effect-site concentration was reduced by 1.5 ng/mL from skin closure until tracheal extubation.

All patients received intravenous patient-controlled analgesia (IV PCA) with fentanyl initiated at the end of surgery. At the discretion of the attending anesthesiologist, one of two regimens was used: (1) fentanyl 1500–3000 μg in a total volume of 100 mL, with a basal rate of 1 mL/h, a bolus dose of 1 mL, and a 15 min lockout; or (2) fentanyl 1700–2000 μg in a total volume of 150 mL, with no basal infusion, a bolus dose of 1 mL, and a 10 min lockout.

RSB was performed after anesthesia induction and before surgical incision. A 23-gauge Quincke needle (TaeChang Industrial, Gongju, Republic of Korea) was advanced under real-time US guidance using a NextGen LOGIQ e console (GE Healthcare, Madison, WI, USA), and 20 mL of 0.375% ropivacaine was injected bilaterally between the rectus abdominis muscle and its posterior sheath, for a total volume of 40 mL.

Postoperative pain was assessed using the 11-point numerical rating scale (NRS; 0 = no pain, 10 = worst imaginable pain). In the PACU, NRS was recorded immediately on arrival, whenever the patient reported pain, and before discharge. The maximum and minimum NRS scores recorded during the PACU stay were used for analysis. For an NRS of 4 or higher, rescue analgesics were administered at the discretion of the attending anesthesiologist and comprised IV fentanyl (25, 40, or 50 μg per dose, up to two or three doses).

When nausea or vomiting occurred at any point during the PACU stay, IV ramosetron 0.3 mg or ondansetron 4 mg was administered at the attending anesthesiologist’s discretion. Rescue antiemetic administration was tracked continuously throughout the PACU stay. In accordance with the institutional PACU protocol, PONV was formally documented at two fixed time points: on arrival to the PACU and at PACU discharge.

On the general ward, NRS was assessed every 4 h (six times daily). IV ketorolac 30 mg was administered every 8 h as scheduled analgesia regardless of NRS, and IV PCA with fentanyl was continued. When the NRS remained ≥4 despite scheduled ketorolac and ongoing PCA, IV tramadol 50 mg was given as the first-line rescue analgesic, followed by IV pethidine (meperidine) 25 mg as the second-line rescue if pain persisted.

### 2.3. Outcomes

The primary outcome was length of hospital stay, defined as the number of calendar days from the day of surgery to the day of discharge. Secondary outcomes included the maximum and minimum NRS scores recorded in the PACU; the weight-adjusted total dose of IV fentanyl (μg/kg) administered as rescue opioid analgesia in the PACU, exclusive of fentanyl delivered through IV PCA; the administration of rescue antiemetics during the PACU stay; the occurrence of PONV at PACU admission and at discharge; and the requirement for rescue analgesics on the general ward on each of POD 0 through 2.

### 2.4. Data Collection

Patient demographics retrieved from the electronic medical records included age, body weight, height, and American Society of Anesthesiologists (ASA) physical status classification. Intraoperative variables comprised duration of surgery (min) and total intraoperative fluid input (mL). Intraoperative urine output was not collected as a study variable because urinary catheterization was performed after surgical draping in a subset of patients, precluding reliable intraoperative urine output monitoring by the anesthesia team in those cases; consequently, complete urine output data were not available across the cohort. Fluid management was not protocolized to a specific urine output or fluid balance target, and intraoperative fluid administration reflected the attending anesthesiologist’s clinical judgment. Postoperative outcomes consisted of the maximum and minimum NRS scores during the PACU stay, weight-adjusted total dose of fentanyl (μg/kg) administered in the PACU, rescue analgesic administration on the general ward, and length of hospital stay (days).

### 2.5. Statistical Analysis

Categorical variables are presented as counts with percentages, *n* (%). Continuous variables are presented as median with interquartile range [Q1, Q3], after assessing the normality of continuous variables with the Shapiro–Wilk test. Because the primary outcome and most continuous variables were non-normally distributed, between-group comparisons of continuous variables were performed using the Mann–Whitney U test. Categorical variables were compared using the chi-square test or, when the expected cell count was less than 5 in more than 20% of cells, Fisher’s exact test. As measures of effect size, the Hodges–Lehmann location shift estimate with 95% confidence intervals (CI) was calculated for continuous outcomes, the median difference with 95% CI derived from the Wilcoxon rank-sum test for integer-valued outcomes (NRS scores and LOS), and the risk difference with 95% CI using the Newcombe score method for binary outcomes. To assess the independent association between RSB and LOS, unadjusted and adjusted quasi-Poisson regression models were constructed. Quasi-Poisson regression was selected on the basis of underdispersion confirmed in the LOS data (dispersion parameter φ = 0.363). The adjusted model included age, body mass index, ASA physical status, and duration of surgery (per 30 min increment) as covariates. Results are expressed as incidence rate ratios (IRR) with 95% CI. Two-sided *p* values < 0.05 were considered statistically significant. All analyses were performed using R version 4.3.3 (R Foundation for Statistical Computing, Vienna, Austria).

## 3. Results

**Figure 1:**
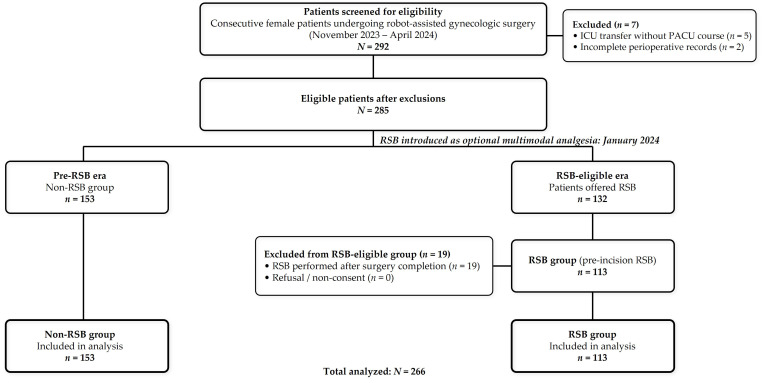
Study flow chart.

A total of 292 consecutive patients were screened for eligibility. Seven patients were excluded: five were transferred directly to the intensive care unit without completing a PACU course, and two had incomplete perioperative records, leaving 285 eligible patients. Of 132 patients in the RSB-eligible era, 19 were further excluded because RSB was performed after surgical completion rather than before incision, and no patient actively refused RSB after preoperative consultation. The final analytic cohort comprised 266 patients: 113 in the RSB group and 153 in the non-RSB group (Figure 1). The two groups were comparable with respect to age, body mass index, ASA physical status classification, duration of surgery, and total intraoperative fluid input (Table 1).

Postoperative outcomes are summarized in Table 2. In the PACU, the weight-adjusted total dose of rescue IV fentanyl was significantly lower in the RSB group than in the non-RSB group (median, 0.86 [IQR, 0.68–1.38] vs. 1.17 [0.85–1.69] μg/kg; *p* < 0.001). Fewer patients in the RSB group required rescue antiemetics in the PACU (4 (3.5%) vs. 18 (11.8%); *p* = 0.029). Although the differences did not reach statistical significance, the occurrence of PONV tended to be lower in the RSB group, both at PACU admission (0 (0.0%) vs. 4 (2.6%); *p* = 0.222) and at PACU discharge (3 (2.7%) vs. 6 (3.9%); *p* = 0.824).

On the general ward, fewer patients in the RSB group received rescue analgesics (tramadol and/or pethidine) beyond scheduled analgesia on POD 0 (59 (52.2%) vs. 105 (68.6%); *p* = 0.009) and POD 1 (15 (13.3%) vs. 52 (34.2%); *p* < 0.001). The between-group difference on POD 2 did not reach statistical significance (25 (22.1%) vs. 46 (30.1%); *p* = 0.191). Length of hospital stay was significantly shorter in the RSB group (median, 3 [IQR, 3–3] vs. 4 [3–5] days; *p* < 0.001).

RSB was independently associated with shorter LOS after adjustment for age, body mass index, ASA physical status, and duration of surgery (adjusted IRR 0.78, 95% CI 0.72–0.85; *p* < 0.001) on quasi-Poisson regression analysis (Table 3). Among the covariates, ASA physical status III (vs. I) and longer duration of surgery (per 30 min increment) were also independently associated with longer LOS.

## 4. Discussion

In this single-center retrospective cohort study of 266 patients undergoing robot-assisted gynecologic surgery, preoperative US-guided RSB was associated with a shorter LOS and a reduction in PACU rescue fentanyl consumption compared with a historical non-RSB cohort. The association with LOS persisted after adjustment for age, body mass index, ASA physical status, and surgical duration in a quasi-Poisson regression model. NRS scores in the PACU did not differ between the groups despite the lower opioid requirement, consistent with an opioid-sparing rather than a pain-eliminating effect of RSB. In addition, RSB appeared to lower the requirement for PACU rescue antiemetics and ward rescue analgesics through POD 1, although the multiplicity of secondary outcomes and the retrospective design preclude definitive conclusions about these endpoints.

The one-day reduction in median LOS observed in the RSB group may be clinically meaningful within the framework of ERAS, in which adequate pain control is considered to facilitate early ambulation, oral intake, and discharge readiness [5,6], though causality cannot be established from a retrospective design. Comparable reductions in LOS following RSB have been reported across diverse abdominal surgical settings: a randomized trial in patients undergoing open abdominal aortic surgery demonstrated a one-day shorter median LOS (5 vs. 6 days) with RSB [11], a retrospective study of single-incision laparoscopic appendectomy reported a 17 h reduction [12], and an early observational study in gynecologic surgery similarly described a one-day shorter ward stay in patients receiving RSB [13]. In contrast, a randomized trial comparing RSB with spinal anesthesia after laparoscopic donor nephrectomy found no significant difference in time to discharge readiness [14], suggesting that the magnitude of any LOS benefit may depend on the underlying surgical population, the comparator analgesic regimen, and institutional discharge practices. Within the context of robot-assisted gynecologic surgery, where minimally invasive techniques and early-recovery pathways are increasingly the norm [3,15], even a one-day reduction in LOS carries appreciable implications for patient experience, healthcare resource utilization, and bed availability.

The reduction in PACU rescue fentanyl consumption without a corresponding change in NRS scores is best interpreted as an opioid-sparing rather than a pain-eliminating effect, a pattern repeatedly observed across the regional anesthesia literature [8,9]. This finding aligns with the established pharmacology and analgesic profile of RSB. The block deposits local anesthetic between the rectus abdominis muscle and the posterior rectus sheath, where it acts on the anterior cutaneous branches of the lower thoracic intercostal nerves (T7–T12) to provide somatic analgesia of the periumbilical region and midline abdominal wall [16]. Because the umbilicus is the principal entry site for the camera port in robotic gynecologic surgery, this sensory distribution is anatomically well matched to the typical pattern of postoperative wound pain in this population. The temporal pattern of analgesic benefit observed in our study—pronounced through POD 1 and dissipating by POD 2—aligns with the pharmacokinetics of ropivacaine, which has an absorption half-life of approximately 4–6 h and produces a clinically relevant sensory block lasting 8–14 h when administered for truncal nerve blocks [17,18,19]. Our observations are concordant with multiple randomized trials of RSB across abdominal surgical populations, which have collectively demonstrated reduced opioid consumption in the early postoperative period after laparoscopic gynecologic surgery [7,20,21,22], laparoscopic-assisted colorectal surgery [23], and abdominal-midline procedures [24], including in comparison with epidural anesthesia [25]. The reduced incidence of rescue antiemetic use observed in our cohort likely reflects this opioid-sparing effect, as opioids are a well-recognized risk factor for PONV [26,27]. In comparison with the only prior report of RSB in robot-assisted gynecologic surgery—a single-arm case series of 20 patients in which all participants received postoperative RSB without a comparator group [10]—the present study, with a substantially larger cohort and an explicit between-group comparison, provides the first comparative evidence that preoperative RSB is associated with reduced opioid requirements in this specific surgical population. Nonetheless, the findings of the present study should be interpreted with caution, given its retrospective and non-randomized nature, and should be considered hypothesis-generating rather than confirmatory.

With respect to safety, no episodes of local anesthetic systemic toxicity were observed in this cohort. All blocks were performed under real-time US guidance using an incremental injection technique. The total ropivacaine dose of 150 mg (20 mL of 0.375% per side) does not exceed the weight-based maximum of 3 mg/kg in patients weighing 50 kg or more; however, 20 of 113 patients (17.7%) in the RSB group weighed less than 50 kg, in whom the administered dose theoretically exceeded this threshold. Despite this, no clinical signs or symptoms of local anesthetic systemic toxicity were observed in any patient, which is consistent with accumulating evidence that ropivacaine, owing to its lower lipid solubility relative to bupivacaine, carries a more favorable cardiovascular safety margin [28,29], and that US-guided truncal blocks are associated with substantially lower peak plasma concentrations than major nerve blocks in highly vascularized regions [29]. Nevertheless, weight-based dose adjustment should be considered in future prospective studies of RSB, particularly in low-body-weight patients.

Several limitations should be acknowledged. First, owing to the non-randomized design, unmeasured baseline differences between the RSB and non-RSB groups cannot be excluded. Although RSB was introduced as an optional technique, no patient declined the block after preoperative consultation, so cohort assignment was determined by surgical era rather than individual patient preference; this minimizes selection bias from patient self-selection, but does not guarantee balance in unmeasured prognostic factors, as would be expected with randomization. Baseline demographic and intraoperative characteristics were nonetheless comparable between the groups. Second, beyond static between-group differences, the era-based design is susceptible to temporal confounding: because the RSB and non-RSB cohorts were drawn from sequential time periods, any unmeasured secular trend in perioperative care could be misattributed to RSB. To mitigate this, we confirmed that the three operating surgeons, the institutional anesthesia and analgesia protocols, and the discharge criteria remained unchanged throughout the study period; nevertheless, residual temporal confounding cannot be fully excluded. Third, the specific gynecologic procedure type (e.g., hysterectomy, myomectomy, sacrocolpopexy) was not consistently retrievable from the structured electronic medical record fields available for this analysis and was therefore not included as a covariate; although duration of surgery, a partial proxy for surgical complexity, was adjusted for in the multivariable model and RSB remained independently associated with LOS, residual confounding from differences in procedure mixes cannot be excluded. Fourth, analyses of rescue analgesic use on the general ward were restricted to POD 0 through POD 2 because more than half of the RSB group had been discharged by POD 3, which would have introduced informative censoring; consequently, the durability of any analgesic benefit beyond POD 2 cannot be evaluated. Fifth, fentanyl administered through PCA was not included in the opioid consumption analysis, and the magnitude of any RSB effect on PCA-delivered fentanyl is unknown. Taken together, these limitations indicate that the present findings should be interpreted as preliminary and hypothesis-generating. Although the consistency of the observed associations across multiple outcomes and the robustness of the LOS finding in multivariable analysis are encouraging, adequately powered randomized controlled trials are necessary to establish a causal effect, to confirm the magnitude of any LOS benefit, to define the optimal local anesthetic agent, volume, and timing, and to formally characterize the safety profile of RSB in this specific surgical population.

## 5. Conclusions

In this retrospective cohort study of patients undergoing robot-assisted gynecologic surgery, preoperative US-guided RSB was associated with reduced PACU rescue opioid and antiemetic requirements, fewer ward rescue analgesic administrations during the early postoperative period, and a one-day shorter median LOS, despite comparable NRS scores between the groups. The LOS benefit persisted after multivariable adjustment, suggesting it is not attributable to measured confounders alone. These findings are consistent with an opioid-sparing effect of RSB; however, given the retrospective, non-randomized design and the multiplicity of outcomes assessed, they should be considered hypothesis-generating. Prospective randomized trials are warranted to confirm the observed benefits and to assess safety.

## Figures and Tables

**Table 1:** Patient demographics and intraoperative characteristics.

	RSB (*n* = 113)	Non-RSB (*n* = 153)	Total (*n* = 266)	*p*-Value
Age, years	44 [37, 51]	43 [38, 52]	44 [38, 52]	0.893
Height, cm	160.9 [157.5, 164.6]	160.1 [156.9, 163.6]	160.4 [157.1, 164.2]	0.152
Weight, kg	59.0 [54.0, 66.3]	59.0 [54.1, 71.8]	59.0 [54.0, 67.2]	0.491
Body mass index, kg/m^2^	22.6 [20.3, 25.7]	22.8 [21.3, 26.4]	22.8 [21.0, 26.0]	0.143
ASA-PS				0.524
I	5 (4.4%)	12 (7.8%)	17 (6.4%)	
II	106 (93.8%)	138 (90.2%)	244 (91.7%)	
III	2 (1.8%)	3 (2.0%)	5 (1.9%)	
Duration of surgery, min	143 [103, 177]	134 [106, 164]	135 [105, 167]	0.715
Total intraoperative fluid, mL	1200 [1000, 1550]	1250 [900, 1500]	1225 [950, 1550]	0.445

Data are presented as median [interquartile range] or *n* (%). Between-group comparisons were performed using the Mann–Whitney U test for continuous variables (age, height, weight, body mass index, duration of surgery, total intraoperative fluid) and Fisher’s exact test for categorical variables (ASA-PS). RSB, rectus sheath block; ASA-PS, The American Society of Anesthesiologists Physical Status.

**Table 2:** Comparison of postoperative outcomes.

	RSB (*n* = 113)	Non-RSB (*n* = 153)	Total (*n* = 266)	Group Difference (95% CI)	*p*-Value
PACU					
Total Fentanyl, μg/kg	0.86 [0.68–1.38]	1.17 [0.85–1.69]	0.94 [0.77–1.61]	−0.25 (−0.42, −0.09)	<0.001 *
Maximum NRS	5 [4–6]	5 [4–7]	5 [4–6]	0 (−1, 0)	0.219
Minimal NRS	2 [1–3]	2 [2–3]	2 [2–3]	−0.5 (−1, 0)	0.001 *
Rescue Antiemetics	4 (3.6%)	18 (11.8%)	22 (8.3%)	−8.3% (−14.9, −1.7)	0.029 *
PONV at admission	0 (0.0%)	4 (2.6%)	4 (1.5%)	−2.6% (−6.5, 0.7)	0.222
PONV at discharge	3 (2.7%)	6 (3.9%)	9 (3.4%)	−1.3% (−6.1, 4.0)	0.824
Analgesic Requirements on the General Ward					
POD 0	59 (52.2%)	105 (68.6%)	164 (61.7%)	−16.4% (−28.0, −4.6)	0.009 *
POD 1	15 (13.3%)	52 (34.2%)	67 (25.3%)	−20.9% (−30.5, −10.8)	<0.001 *
POD 2	25 (22.1%)	46 (30.1%)	71 (26.7%)	−7.9% (−18.3, 2.9)	0.191
LOS, days	3 [3–3]	4 [3–5]	3 [3–4]	−1 (−1, 0)	<0.001 *

Data are presented as median [interquartile range] or *n* (%). Between-group comparisons were performed using the Mann–Whitney U test for continuous variables (total fentanyl, NRS, LOS) and Fisher’s exact test for binary variables (rescue antiemetics, PONV, analgesic requirements on POD 0–2). Group difference represents the Hodges–Lehmann location shift estimate with 95% CI for total fentanyl; the median difference with 95% CI derived from the Wilcoxon rank-sum test for integer-valued outcomes (NRS, LOS); and the risk difference with 95% CI calculated using the Newcombe score method for binary outcomes; all expressed as RSB minus non-RSB. * *p* < 0.05. RSB, rectus sheath block; NRS, numeric rating scale; PACU, post-anesthesia care unit; PONV, postoperative nausea and vomiting; POD, postoperative day; LOS, length of hospital stay.

**Table 3:** Unadjusted and adjusted incidence rate ratios for length of hospital stay.

	Unadjusted IRR (95% CI)	*p*-Value	Adjusted IRR (95% CI)	*p*-Value
RSB (vs. non-RSB)	0.79 (0.72, 0.86)	<0.001	0.78 (0.72, 0.85)	<0.001
Age (per year)	1 (1, 1)	0.813		
Body mass index (per kg/m^2^)	1.01 (1, 1.02)	0.126		
ASA-PS II (vs. I)	0.89 (0.76, 1.05)	0.163		
ASA-PS III (vs. I)	1.84 (1.4, 2.4)	<0.001	1.75 (1.37, 2.22)	<0.001
Duration of surgery (per 30 min)	1.04 (1.02, 1.06)	<0.001	1.04 (1.02, 1.06)	<0.001

Unadjusted and adjusted quasi-Poisson regression models were constructed on the basis of underdispersion confirmed in the LOS data. *p*-values were derived from quasi-likelihood-based Wald tests. IRR < 1 indicates shorter LOS; IRR > 1 indicates longer LOS. IRR, incidence rate ratio; CI, confidence interval; RSB, rectus sheath block; ASA-PS, The American Society of Anesthesiologists Physical Status.

## Data Availability

The data presented in this study are available on request from the corresponding author.

## Abbreviations

The following abbreviations are used in this manuscript:

ASA	American Society of Anesthesiologists
ERAS	Enhanced Recovery After Surgery
IV	Intravenous
LOS	Length of hospital stay
NRS	Numerical rating scale
PACU	Post-anesthesia care unit
PCA	Patient-controlled analgesia
POD	Postoperative day
PONV	Postoperative nausea and vomiting
RSB	Rectus sheath block
US	Ultrasound
